# Susceptibility of Multiple Primary Cancers in Patients With Head and Neck Cancer: Nature or Nurture?

**DOI:** 10.3389/fonc.2019.01275

**Published:** 2019-11-21

**Authors:** Wei-long Zhang, Zhuo-li Zhu, Mei-chang Huang, Ya-Jie Tang, Ya-ling Tang, Xin-hua Liang

**Affiliations:** ^1^State Key Laboratory of Oral Diseases, National Clinical Research Center for Oral Diseases, West China Hospital of Stomatology, Sichuan University, Chengdu, China; ^2^State Key Laboratory of Microbial Technology, Shandong University, Qingdao, China

**Keywords:** head and neck cancer, multiple primary cancer, field cancerization, cancer-associated fibroblasts, genetic factor, biomarker

## Abstract

Multiple primary cancers (MPCs) are major obstacles to long-term survival in head and neck cancer (HNSCC), however, the molecular mechanism underlying multiple carcinogenesis remains unclear. “Field cancerization” is a classical theory to elaborate the malignant progression of MPCs. Apart from environmental and immune factors, genetic factors may have great potential as molecular markers for MPCs risk prediction. This review focuses on inherited and acquired gene mutations in MPCs, including germ-line mutation, single-nucleotide polymorphism, chromosomal instability, microsatellite instability and DNA methylation. And definition and prognosis of MPCs have also been discussed. These may pave the way for the early detection, prevention and effective treatment of MPCs in HNSCC.

## Introduction

Head and neck squamous cancer (HNSCC) ranks sixth among the most prevalent malignancies in the world (1). It is estimated that HNSCC accounts for 64,690 new cases and 13,740 deaths in the United states in 2018 (2). Although significant improvements have been made in the therapeutic modalities, the prognosis of HNSCC remains stagnant in the past decades, with a 5-year survival of only 50% (3). The dismal prognosis has always been attributed to local recurrences, distant metastasis, and development of multiple primary cancers (MPCs).

MPCs are defined as two or more primary cancers occurring in an individual synchronously or metachronously, neither extensions, recurrences nor metastases of each other (International Agency for Research on Cancer), which accounts for approximately one-third of deaths in HNSCC (4, 5). MPCs are also named as second primary malignancies (SPMs), secondary primary tumors (SPTs), second primary cancers (SPCs), and multiple primary tumors (MPTs) (6). Several risk factors, including smoking exposure, alcohol consumption, human papilloma virus (HPV), and hepatitis C virus (HCV), have been suggested to be associated with the development of MPCs (7–10). However, researches revealed that a major proportion of MPCs could not be fully explained by these environmental factors, and genetic factors, such as single-nucleotide polymorphism (SNP), chromosomal instability (CIN), microsatellite instability (MSI), and epigenetic alterations, may contribute to the susceptibility of MPCs in HNSCC (11).

MPCs have been considered to be a clinical quandary both in diagnosis and treatment. It is challenging for the oncologists to distinguish MPCs from a metastasis or local recurrence merely on the basis of clinical and pathological information. Furthermore, this classification leaves a profound effect on the choice of treatment as well as patients' prognosis. Recently, there are a number of novel screening strategies for MPCs prediction, such as genetic markers, and the role of genetic alterations in the development of MPCs after index HNSCC were not fully elucidated (12). Here, we conducted this review to summarize the mechanisms and relevant genetic alterations of MPCs, which might provide benefit in the detection, prevention, and treatment of MPCs in HNSCC.

## Definition and Prognosis of MPCs

In 1932, Warren and Gates published the classical clinical criteria of SPT, being (1) each of the malignancies must have been verified by histologic examination, (2) the malignancies must be anatomical separate by normal mucosa, and (3) exclude the possibility that the second malignancy represents a metastasis of the index tumor (13). However, this clinical definition carries the risk of misclassification and inability to differentiate between an SPT, a recurrence and a metastasis (14). For example, what distance should lie between the malignancies? How to define the normal mucosa, by naked eye or histologic examination? How to distinguish the SPT from a metastasis or a recurrence? To solve this dilemma, Braakhuis et al. proposed a new classification on the basis of molecular profiles of the tumors and the genetically altered mucosal field between the tumors (14). Tumors displaying distinct molecular profiles or sharing a common pattern attributed to chance are defined as SPTs, while those possessing similar molecular aberration are defined as local recurrences (14).

It is widely accepted that MPCs are the leading obstacle to long-term survival among HNSCC patients (15). According to a retrospective study conducted by Shiga et al., patients with synchronous SPMs displayed a poorer 5-year overall survival rate than those with metachromous SPMs in Japan (16). In line with this result, Bugter et al. claimed that the 5-yearsurvival rate for synchronous and metachronous primary cancer patients was 25 and 85%, respectively (17). The higher proportion of high-stage tumor in the synchronous primary cancer patients and unadjusted treatment protocol may account for this discrepancy.

However, accumulating evidence has demonstrated that HPV-positive HNSCC patients were accompanied by a decreased risk for SPT than HPV-negative HNSCC patients (18–20). HPV has been established as an emerging carcinogen in a subset of HNSCCs, particularly in the oropharynx (21). HPV-positive HNSCCs differ from HPV-negative HNSCCs induced by tobacco and alcohol epidemiologically, clinically, and biologically (22–24). The putative reasons for this phenomenon are as follows: (1) HPV-positive HNSCCs exhibited higher sensitivity to radiotherapy and chemotherapy (25); (2) HPV-positive HNSCCs often arise in an environment with lower exposure to tobacco and displayed fewer smoking-related genetic abnormalities, which is less associated with smoking-related SPTs (26–28); (3) Saito et al. suggested that field cancerization effect would not be observed in HPV-positive HNSCCs, since HPV viral DNA integration was limited to the cancerous tissue (29). However, the concrete mechanisms between HPV-positive HNSCCs and SPTs are remained to be elucidated in the future.

The dismal clinical outcome of MPCs in HNSCC emphasizes on the importance of early diagnosis and prevention. These genetic alterations could serve as molecular makers to guide the early diagnosis, prevention and treatment of HNSCC patients in several aspects. Firstly, these molecular markers could be readily obtained by the primary tumor samples without bringing additional invasion to patients (30). Secondly, genetic markers could select the high-risk individuals for MPCs, which should be under strict cancer surveillance and proper preventive procedures. Given the discrepancies between HPV-positive and HPV-negative HNSCC, Jain et al. proposed that future screening procedures for MPCs may be adjusted by HPV and smoking status (31). Thirdly, in-depth understanding of the role of these molecular markers in MPCs may pave the way for targeted gene therapies. In addition, genetic markers could be utilized to distinguish MPCs from a local recurrence or a metastasis. For example, Mercer et al. showed that microsatellite PCR facilitated the discrimination between second primary cancer and metastatic HNSCC (32). Daher et al. employed combined HPV typing and TP53 mutational profiling successfully identified the accurate origin of lung tumors in 32 HNSCC patients, in which only 13 cases were diagnosed correctly on the basis of clinical and morphological data alone (33). Apart from physical and pathological information, genetic profiles analysis could be an effective tool to distinguish MPCs from a recurrence. Gasparotto et al. suggested that 3 clinically diagnosed recurrences and 2 lung lesions were actually MPTs by comparing the p53 mutation status of primary tumors and corresponding recurrences/metastases in HNSCC patients (34). Microsatellite analysis indicated that 6 tumors showing clonally-related patterns with primary tumors were recurrences, while 17 tumors with clonally-unrelated patterns were SPTs in 23 HNSCC patients with genetic changes (35).

## Mechanism of MPCs

**Figure 1:**
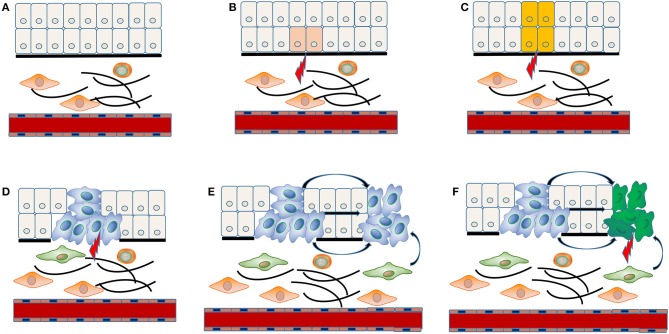
“Field cancerization” theory in the MPCs of HNSCC. The stem cell receives first genetic hit **(A)**, and gives rise to a patch with genetically altered daughter cells **(B)**. Then a patch develops into a field by expanding in a lateral direction under the indduction of a second genetic hit **(C)**. Additional genetic alterations take place and convert the field to an overt carcinoma **(D)**. The implantation, intraepithelial migration or sub-mucosal spread of primary cancer cells lead to the development of a SPT with monoclonal origin **(E)**, while the final genetic hit induces the occurrence of a SPT with polyclonal origin **(F)**.

The concrete molecular mechanism underlying multiple carcinogenesis remains unclear. “Field cancerization” theory has often been applied to explain the occurrence of MPCs (Figure 1). The stem cell receives one (or more) genetic hit (Figure 1A), probably a mutation of *p53* gene, and gives rise to a patch with genetically altered daughter cells [(36); Figure 1B]. Then a subsequent genetic alteration induces the patch to spread in a lateral direction and substitutes the normal epithelial cells to form a field [(36); Figure 1C]. As the field expands at the expense of normal epithelial cells, additional genetic alterations take place and promote the progression from field to an overt carcinoma (Figure 1D). The second tumor of monoclonal origin develops by implantation, intraepithelial migration or sub-mucosal spread of primary cancer cells (Figure 1E), while the polyclonal second tumor forms under the induction of final genetic hit [(14, 37); Figure 1F].

**Figure 2:**
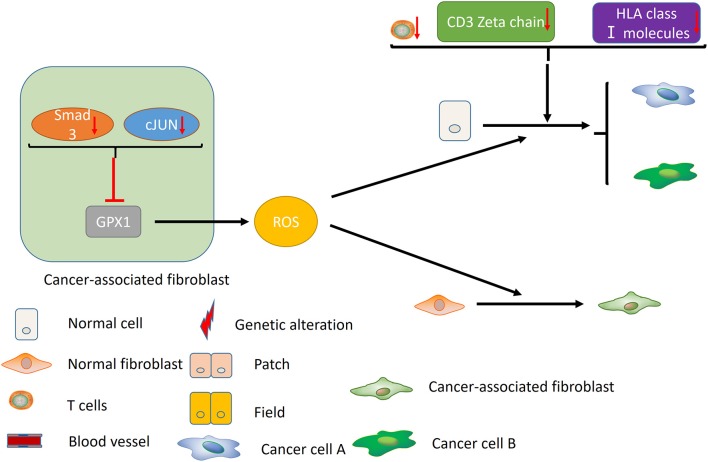
Immune factors and CAFs contribute to the development of MPCs. Decreased T-cell numbers, CD3 zeta chain and HLA class I molecules may promote the development of MPCs by inducing immunosuppression. In the CAFs, reduced expression of Smad3 and cJUN suppress the activity of GPX1, leading to the elevation of extracellular hydrogen peroxide. High hydrogen peroxide level in the microenvironment induces the conversion of normal fibroblast to CAF phenotype, and promotes the occurrence of MPCs.

Failure of immune surveillance also contributed to the occurrence of SPTs in HNSCC (Figure 2). Patients with decreased T-cell numbers in the circulation were predisposed to infections, disease recurrence, or a second malignancy (38). Kuss et al. reported that CD4+ and CD8+ T cells were significantly reduced in the SPT group relative to normal control group in HNSCCs (38). And patients with recurrences or SPTs showed a 25% lower number of CD4+ T cells than those with primary disease (38). The TCR associated CD3 zeta chain plays a critical role in the signal transduction of T-cell activation, the absence of which impairs T-cell signaling and consequently leads to immune dysfunction (39). Kuss et al. concluded that individuals with SPTs or recurrences exhibited lowest zeta-chain expression, which might exert long-lasting negative effects on the anti-tumor immune response (40). Decreased expression of HLA class I molecules is considered to be an effective strategy for malignant cells to evade host immunosurveillance (41). Grandis et al. suggested that the number of HLA allelic loss increased the risk of developing a new primary tumor (41). Collectively, decreased T-cell numbers, CD3 zeta chain and HLA class I molecules may be associated with the development of SPT, which may provide new opportunities for cancer immunotherapy in HNSCC.

In addition, cancer-associated fibroblasts (CAFs) may play an unneglectable role in the development of field cancerization [(42); Figure 2]. Ge et al. proposed that migratory cancer-associated fibroblasts (CAFs), also named myofibroblast, may appear beneath the cluster of genetic altered epithelial cells, and ultimately lead to the malignant transformation of these cells (42). Angadi et al. demonstrated that myofibroblasts were present in the stroma around the oral squamous cell carcinoma (OSCC) cell as well as the connective tissue below the histologically normal mucosa adjacent to OSCC by immunochemistry, which validates Ge's hypothesis further (43). Chan et al. indicated that cancer-associated fibroblasts promoted field cancerization by elevating the expression of reactive oxygen species (ROS) in the microenvironment (44). CAFs from squamous cell carcinoma reduced the expression of Smad3 and cJUN to suppress the activity of glutathione peroxidase 1 (GPX1), one key enzyme affecting hydrogen peroxide detoxification. Suppression of GPX1 leads to elevation of extracellular hydrogen peroxide, which facilitates the conversion of normal fibroblast to CAF phenotype, and promotes the tumor-forming capacity and invasiveness. Till now, there is no more available evidences on the role of tumor microenvironment in MPCs, including macrophages, myeloid-derived suppressor cells (MDSCs) and etc., which warrants further investigation in the future.

## MPCs and Inherited Mutations

### MPCs and Germ-Line Mutations

Germ-line mutation of tumor suppressor genes has been considered to be a potential driver of MPCs. *p53* gene, known as the “the guardian of the genome,” is an well-known tumor suppressor gene which has been involved in the cell cycle control and DNA repair (45). It is estimated that *p53* gene was mutated in approximately 50% of HNSCC patients (46). In 1999, Gallo and his colleagues employed polymerase chain reaction single-strand conformation polymorphism (PCR-SSCP) analysis and DNA sequencing to examine the *p53* germ-line mutations in 24 HNSCC patients who developed MPCs and their first-degree relatives. As a consequence, only one missense mutation in exon 6 as well as two same-sense mutations in exon 6 and 8 have been detected (47). So the authors proposed that *p53* gene might not be the only target responsible for the multiple genetic alterations of field cancerization (47).

*CDKN2A*, a tumor suppressor gene exerting an important role in the regulation of cell cycle, is associated with the occurrence of HNSCC. Cabanillas et al. proposed that germ-line mutations of *CDKN2A* gene may serve as a common feature of HNSCC (30). However, Jefferies et al. screened full coding sequence of *CDKN2A* gene and failed to detect any germ-line mutations in 40 HNSCC patients with a SPC, which suggested that germ-line mutations of *CDKN2A* contributed less to the susceptibility of MPCs (48).

Mismatch genes, such as *hMLHI*, contribute greatly to the MPCs of gastrointestinal cancers. Nevertheless, its germ-lime mutations don't seem to be an major event in the carcinogenesis of HNSCC or MPTs (49). Piccinin et al. analyzed the mutations of *hMLHI* gene by PCR-SSCP and sequencing in 67 HNSCC patients, 22 MPTs and 45 controls, and no somatic or germ-lime mutations of hMLHI have been identified (49).

Based on the above, it seems that germ-line mutations of certain tumor suppressor genes and DNA repair genes, including *p53, CDKN2A*, and *hMLHI*, exert a minor influence on the genetic predisposition of MPCs after index HNSCC.

### MPCs and SNPs

It is estimated that SNP accounts for up to 90% of genetic variability (50). Researchers have demonstrated that genetic polymorphisms of various genes were correlated with the risk of MPCs in HNSCC. Based on their functions, these genes could be classified into four categories: tumor suppressor genes, oncogenes, DNA repair genes and carcinogen metabolism-related genes, which were listed in detail as follows.

#### SNPs of Tumor Suppressor Genes

*p53* makes a substantial contribution to the regulation of cell cycle, cellular apoptosis and anti-cancer properties (45). *p53* mutations have been shown to be correlated with the occurrence of primary HNSCC as well as SPMs (51). The polymorphism of *p53* commonly occurred at the codon 72, with a substitution of proline for arginine, which might promote the carcinogenesis by disturbing the apoptosis process and cell cycle (52). Several studies indicated that *p53* codon 72 polymorphism may play a minor role in the development of HNSCC (53, 54). However, Li et al. showed that patients with *p53* 72Arg/Pro and the combined *p53* 72Arg/Pro + Pro/Pro genotypes exhibited a significantly greater risk of SPMs in a cohort of 1271 HNSCC patients, compared with *p53* 72Arg/Arg genotype (51). The authors speculated that *p53* codon 72 polymorphism may influence the SPM risk by modifying the response to DNA-damaging treatments (51). Despite the inherited limitations of patient selection and clinical outcome collection, these results indicated that *p53* codon 72 polymorphism could serve as a genetic marker to evaluate the risk of SPMs in HNSCC.

Functionally, tumor suppressor gene *p14* and *p73* belong to the *p53*-related gene family. *p14*^*ARF*^ gene maps to 9p21 and encodes proteins of *p16*^*INK*4*a*^ and *p14*^*ARF*^, thus playing an important role in maintaining genomic stability via *p53* and *Rb* pathways (6). Direct interaction between *p14*^*ARF*^ and *MDM2* impedes the protesomal degradation of p53, resulting in an abnormal cell cycle regulation and cellular apoptosis (55). Genetic Alterations of *p14*^*ARF*^ have been considered to be rare in the development of HNSCC. Gruttgen et al. evaluated the p14 expression with immunochemistry, and concluded that loss of *p14*^*ARF*^ merely occurred in 15 of 100 HNSCC patients (56). Zhang et al. stated that *p14*^ARF^-rs3088440 and rs3731217 polymorphisms were correlated with a moderately increased risk of SPMs in HNSCC (6). Compared with those without *p14*^*ARF*^ variant genotypes, HNSCC patients with both variant genotypes had a 3-fold increased risk for developing SPMs (6). Therefore, *p14*^*ARF*^ polymorphisms could serve as a risk marker for SPMs in HNSCC patients (6).

*p73* displays a similar function as *p53* in the regulation of cell cycle, apoptosis and DNA repair by inducing apoptosis or G1 cell cycle arrest (57). It has been assumed that *p73* compensated for the absence of *p53* induced by mutations (58). Previous studies have revealed that genetic abnormalities of *p73*, such as its G4C14-to-A4T14 polymorphism, were associated the risk of HNSCC (59, 60). Li et al. investigated the role of *p73* G4C14-to-A4T14 polymorphism in SPMs in a cohort of 1384 HNSCC patients, and advocated that patients carrying *p73* GC/AT heterozygotes or the combined *p73* GC/AT+AT/AT genotypes had a significantly lower SPM susceptibility, compared to those with *p73* GC/GC genotype. The *p73* GC/AT+AT/AT genotypes conferred a pronounced protection over SPMs in several subgroups, for example, older patients, men, minorities, ever smoker, and ever drinkers, further supporting the role of *p73* polymorphism as a genetic marker of MPCs in HNSCC patients (61).

*FAS* belongs to the death receptor family and interacts with its ligand, *FASLG*, to modulate the extrinsic apoptosis pathway, cellular homeostasis and immune escape of tumor cells (62, 63). Genetic alterations of the *FAS/FASLG* signaling pathway may lead to immune evasion, thus facilitating tumorigenesis including SPM (64). *FAS*-1377G>A, *FAS*−670A>G, *FASLG*-844C>T, and *FASLG*-124 A>G are four well-known SNPs in the FAS/FASLG signaling pathway. Zhang et al. reported that subjects with both the *FAS*-1377 AA and *FAS*-670 (GG + AG) genotypes were associated with an increased risk of HNSCC, but not for those with *FASLG* variant genotypes (65). But things are really different when it comes to the incidence of MPCs. It has been demonstrated that patients carrying *FAS*-670 AG+GG genotypes or *FASLG*-844 CT+TT genotypes were significantly associated with mounting risk of SPMs compared with the wild-type homozygous genotypes, which makes *FAS* and *FASLG* polymorphisms a potential marker for HNSCC patients at high SPM risk (64). Additionally, the risk of SPM was augmented in a dose-response manner for those with increasing number of risk genotypes (64).

p21 and p27 are two CDK inhibitors which participate in the regulation of DNA repair, cell cycle, and apoptosis (66). It has been demonstrated that SNPs in *p21* and *p27* were associated with risk of HNSCC (67, 68). HNSCC patients carrying *p27* 109 TG/GG, *p21* 98 CA/AA, and *p21* 70 CT/TT variant genotypes had a worse survival and an increased SPM risk than those with *p27*109 TT, *p21* 98 CC, and *p21* 70 CC genotypes, respectively (69). Moreover, patients with *p27* (T109G) and *p21* (C98A and C70T) polymorphisms were 2.4 times more susceptible to develop MPCs than those without variant genotypes (69). These results indicated that *p27* T109G and *p21* (C98A and C70T) polymorphisms seem to modulate the susceptibility of SPMs in HNSCC.

Accumulating evidences have established that SNPs of tumor suppressor genes made a considerable contribution to the formation of MPCs in HNSCC, which makes SNPs of tumor suppressor genes a potential molecular marker to predict the risk of MPCs in HNSCC.

#### SNPs of Oncogenes

Murine double minute 2 (*MDM2*), also known as an E3 ubiquitin ligase, is the central antagonist of the tumor suppressor *p53* (70). MDM2 negatively regulates the activity of *p53* by suppressing its transcriptional activity and promoting its degradation, thus contributing to the carcinogenic process (71). The overexpression and genetic alterations of *MDM2* have been commonly reported in HNSCC (72). Two SNPs in its promoter region, *MDM2*-rs2279744 and *MDM2*-rs937283, may alter MDM2 expression at transcriptional level and subsequently modulate the risk of HNSCC (71). With respect to SPM, Jin et al. reported that *MDM2*-rs2279744 and *MDM2*-rs937283 increased the susceptibility of SPM in HNSCC by 90 and 20%, respectively (73).

Analogous to *MDM2* in structure, murine double minute 4 (*MDM4*) is also a negative regulator of *p53*. Several studies suggested that high *MDM4* expression may substitute for *p53* mutations, and *MDM4* overexpression was a common event in the HNSCC patients (74). Three *MDM4* SNPs, rs11801299G>A, rs1380576C>G, and rs10900598G>T, have been identified in HNSCC patients. Yu et al. proposed that individuals with combined 1-3 risk genotypes of *MDM4* SNPs exhibited significantly increased risk of oropharyngeal cancer (75). In a cohort of 1283 HNSCC patients, Jin et al. concluded that *MDM4*-rs11801299, *MDM4*-rs1380576, and *MDM4*-rs10900598 enhanced the incidence of SPMs in index HNSCC cases by 10, 10, and 40%, respectively (73). Collectively, *MDM2* and *MDM4* polymorphisms may increase the susceptibility of SPMs in HNSCC to some extent, which may improve the precision of risk estimates of SPMs.

#### SNPs of DNA Repair Genes

The MRN complex, composed of *MRE11, RAD50*, and *Nbs1*, plays a critical role in the double-strand break repair and telomere maintenance (76). To evaluate the role of *MRE11* and *RAD50* genes in HNSCC, Ziółkowska-Suchanek et al. conducted a case and control study of 358 HNSCC patients. Their results suggested that common variants of *MRE11* and *RAD50* genes contributed little to the occurrence of HNSCC and SPTs located in the head and neck region (77). *XRCC3*, short for X-ray repair cross-complementing group 3, is another important gene which participates in the double-strand break repair (78). Several studies revealed that *XRCC3* C18067T polymorphism may play a minor role in the etiology of primary HNSCC as well as MPTs. (78, 79).

The X-ray repair cross-complementing group 1 (*XRCC1*) exerts on a vital role in the DNA single-strand break repair pathway (80). *XRCC1* Arg194Trp, *XRCC1* Arg280His, and *XRCC1* Arg399Gln are the three most common examined SNPs in the *XRCC1* gene (81, 82). Lou et al. suggested that *XRCC1* Arg194Trp, *XRCC1* Arg280His, and *XRCC1* Arg399Gln posed limited effect on the HNSCC risk in a meta-analysis with 29 studies (83). Similarly, no significant associations have been presented between *XRCC1* gene SNPs and the incidence of MPT in the HNSCC patients (79).

Apart from double-strand and single-strand repair pathway, the host can protect the genome from damage induced by various environmental carcinogens by means of nucleotide excision repair (NER) pathway (84). Seven SNPs of the *NER* genes involved in the HNSCC are listed as follows: *XPC* Ala499Val, *XPC* Lys939Gln, *XPD* Asp312Asn, *XPD* Lys751Gln, *XPG* His1104Asp, *ERCC1* C8092A, and *XPA* G23A. Zafereo et al. declared that no significant association between aforementioned seven SNPs and the SPM susceptibility, independently or collectively, has been found in a recessive model (84).

All in all, no significant associations between SNPs of DNA repair genes and MPC risk have been found so far. On the basis of above evidences, it is plausible that SNPs of DNA repair genes might not play a major role in the development of MPCs in the HNSCC subjects.

#### SNPs of Carcinogen Metabolism-Related Genes

Glutathione peroxidase I (GPX1), a selenium-dependent enzyme, participates in the detoxification of activated oxygen species (85). Genetic alterations or polymorphism in the coding region of *GPX1* gene might be involved in the development of cancer. A significant correlation has been observed between *GPX1* expression and T-stage as well as index tumor sites in HNSCC patients (86). The *GPX1* polymorphism represents three possible alleles, namely ALA5, ALA6, and ALA7. Jefferies et al. evaluated the association between *GPX1* genetic polymorphisms and HNSCC patients who developed SPTs in a case-control study. A significant difference in allele frequencies of *GPX1* ALA^*^6 and ALA^*^7 was observed between the SPT cases and controls, which indicated that polymorphisms of *GPX1* gene may be a molecular marker for the development of SPTs in HNSCC (85).

*CYP1A1* and *CYP2E1* are two main genes associated with the carcinogen metabolic activation. Rydzanicz et al. reported that HNSCC patients with *CYP1A1* genotype ^*^1/^*^4 and allele ^*^4 represented a 4.1- and 2.6-fold risk of developing MPT, respectively (79). However, no significant correlations has been established between SNPs of *CYP2E1* gene and the incidence of MPT, which suggested a limited role of *CYP2E1* in the susceptibility of MPTs in HNSCC (79).

Glutathione S transferase (GST) plays a critical role in the detoxication and elimination of various carcinogens (87). *GSTM1, GSTT1*, and *GSTM3* gene are three members of the GST family in human (87). Studies reported that the *GSTT1* null genotype and polymorphism in *GSTM3* gene were not correlated with a statistically significant increased risk for SPTs or tobacco-related SPTs (79, 88). Inversely, a significant association was observed between the polymorphism in *GSTM1* gene and development of SPTs or tobacco-related SPTs (88).

N-acetyltransferase 2 (*NAT2*) gene participates in the metabolism of aromatic, heterocyclic amines and hydrazines (89). Evidence from 23 case and control studies indicated that *NAT2* polymorphisms could increase the incidence of HNSCC by 23% and serve as a risk factor of HNSCC in Asians (90). With respect to MPCs, NAT2^*^7B was significantly correlated with an increased risk for SPTs in patients after index HNSCC (79).

According to the available evidences, a significant association has been observed between MPCs and SNPs of carcinogen metabolism-related genes, such as *CYP1A1, GSTM1*, and *NAT2*. However, well-designed, large-scale, multi-center studies are still warranted to verify these conclusions in the future.

## MPCs and Acquired Mutations

### MPCs and CIN

CIN comprises altered DNA copy number and loss or rearrangement of the chromosomes, resulting in the loss or gain of function of certain genes (91). Piccinin et al. evaluated the LOH status at 1p, 3p, 9p, 13q, and 19p. However, no significant differences have been observed between the MPCs group and single cancer group, which suggested that chromosomal instability may not account for the propensity to develop SPMs in the upper aerodigestive tract (49).

### MPCs and MSI

MSI, a major hallmark of genetic instability, originates from deficient DNA mismatch repair (92). It is mostly observed and studied in the hereditary non-polyposis colorectal cancer, which is characterized by MPCs of different organs, such as gastrointestinal, endometrial and urinary tract (93). So, MSI is considered to be a major determinant in the development of MPCs. Piccinin et al. analyzed the MSI on five chromosomes in 67 HNSCC patients, 22 MPCs and 45 controls, and revealed that no significant differences existed between MSI and MPCs cases (49). This implied that except for MSI, other systems concerning the genome integrity might be responsible for the carcinogenesis of HNSCC and tumor multiplicity of the head and neck region (49).

### MPCs and DNA Methylation

DNA methylation is a well-categorized change of epigenetic alterations in tumors, which is capable of silencing the classic tumor suppressor genes (94). DNA methylation could disrupt the tumor suppressor gene function by obstructing its promoter region and impeding the transcriptional process (95). Longo et al. have detected *CCNA1, DCC*, and *TIMP3* hypermethylation in the exfoliated cell samples of HNSCC patients (96). To investigate the relationship between hypermethylation and MPCs in HNSCC, Rettori et al. examined the methylation patterns of 19 genes in 70 HNSCC cases (97), revealing that *CCNA1* and *TIMP3* hypermethylation were significantly connected with formation of SPT in HNSCC. Hypermethylation of *CCNA1* and *TIMP3* might be a promising genetic marker to predict the incidence of SPT in HNSCC subjects, providing the basis for the use of preventive measures and adjuvant treatment.

## Conclusions

The dismal prognosis of HNSCC has always been attributed to the occurrence of MPCs. “Field cancerization,” induced by carcinogens and CAFs, is proposed to elaborate the development of MPCs. Apart from environmental and immune factors, genetic factors may play a major role in the risk of MPCs. In summary, SNPs of tumor suppressor genes, oncogenes and carcinogen metabolism-related genes, together with DNA methylation, may serve as potential molecular markers of MPCs risk (Table 1). SNP chips and next-generation sequencing technology will enables us to access the strength of these “nature” components of MPCs, resulting in early diagnosis and better survival in HNSCC patients. However, there is still a long way to go before the clinical application of these genetic markers. HNSCC is a genetically heterogeneous disease with a wide range of genetic alterations (98), so a panel of genetic markers with the most accuracy and specificity need to be selected. On the other hand, large-scale, well-designed, and multi-center studies are warranted to examine their clinical relevance.

**Table 1:** Genetic factors, genes, and potential biomarkers of multiple primary cancer of HNSCC.

**Factors**	**Genes**	**Potential biomarkers**
Germline mutation	*p53, CDKN2A, hMLHI*	Unidentified
SNP	Tumor suppressor genes: *p53, p1p14, p73*, *FAS/FASLG, p21, p27* Oncogenes: *MDM2, MDM4* DNA repair genes: *MRE11, RAD50, NBN*, *XRCC3, XRCC1, XPC, XPD, XPG, ERCC1, XPA* Carcinogen Metabolism-related genes: *GPX1, CYP1A1, CYP2E1, GSTT1*, GSTM1, *GSTM3, NAT2*	*p53, p1p14, p73, FAS/FASLG, p21, p27* *MDM2, MDM4* Unidentified *GPX1, CYP1A1, GSTM1, NAT2*
CIN MSI Epigenetic alterations	Unidentified Unidentified *CCNA1, DCC, TIMP3*	Unidentified Unidentified *CCNA1, TIMP3*

## Author Contributions

All authors listed have made a substantial, direct and intellectual contribution to the work, and approved it for publication.

### Conflict of Interest

The authors declare that the research was conducted in the absence of any commercial or financial relationships that could be construed as a potential conflict of interest.
